# Clinicodemographic Data of Patients with Behçet’s Disease: Data from a Tertiary Center in Saudi Arabia

**DOI:** 10.2174/0115733971359862250603040950

**Published:** 2025-06-10

**Authors:** Yasser Bawazir, Mohammad Mustafa

**Affiliations:** 1 Department of Medicine Faculty of Medicine, King Abdulaziz University, Jeddah, Saudi Arabia;; 2 Department of Medicine, Rheumatology Unit, University of Jeddah, Jeddah, Saudi Arabia

**Keywords:** Behcet disease, chronic inflammatory vasculitis, silk route disease, demographic factors, high BMI, clinical manifestations, body mass index

## Abstract

**Introduction:**

Behçet’s disease (BD) is a chronic inflammatory vasculitis involving the arteries and veins. This study was driven by the rarity, chronic multisystemic nature, and heterogeneous spectrum of clinical features and geographical distribution. This study aimed to analyze the demographic and clinical characteristics of patients with BD at the King Abdulaziz University Hospital and identify the association between clinical and laboratory findings and disease severity.

**Methods:**

The study was a retrospective core chart review. This study included adult patients who visited the rheumatology clinic of King Abdulaziz University Hospital in Saudi Arabia between 2005 and 2023. The inclusion criteria were age ≥18 years and a diagnosis of Behçet’s disease (BD) based on either the International Criteria for Behçet’s Disease or the International Study Group classification criteria.

**Results:**

In total, 81 patients with BD with almost equal male (51.9%) and female (48.1%) distribution, 75.3% Saudi nationals, mean onset age of 38.48 years, and mean body mass index of 27.57 kg/m^2^ were identified. The most common clinical manifestations were oral ulcerations (56.8%), genital ulcerations (37%), uveitis (24.7%), arthritis (22.2%), skin lesions (13.6%), and deep vein thrombosis (9.88%). Significant differences in high-density lipoprotein, hemoglobin, C-reactive protein, and albumin levels were associated with the age, sex, and nationality of the patients, respectively. Similarly, body mass index was significantly associated with C-reactive protein (*p* = 0.004), alanine aminotransferase (*p* = 0.023), aspartate aminotransferase (*p* = 0.003), and gamma-glutamyl aminotransferase (*p* = 0.034) levels.

**Discussion:**

The observed clinical and demographic patterns align with regional and global data, though a slightly older age at onset and high BMI prevalence were noted. Associations between BMI and inflammatory or hepatic markers suggest a possible metabolic influence on disease activity. Laboratory differences across demographic subgroups emphasize the need for individualized disease assessment. These insights can inform tailored care strategies for BD patients in the Saudi context.

**Conclusion:**

This study demonstrated that there are significant associations between demographic factors, laboratory parameters, and BD activity.

## 1 INTRODUCTION

Behçet’s disease (BD) is a chronic inflammatory vasculitis involving the arteries and veins. It usually begins during the third or fourth decade of life. The clinical domains of patients with BD vary based on demographics, disease course, and treatment responses. These clinical domains can be classified into musculoskeletal, mucocutaneous, ocular, vascular, central nervous system, and gastrointestinal tract manifestations [1]. The disease is particularly prevalent in the countries along the Silk Road and is thus termed the Silk Route disease [2, 3]. The highest prevalence rate was identified in Turkey (80-421 per 100,000 of the population aged ≥12 years) [2]. In Saudi Arabia, the prevalence rate is 19.5 per 100,000 [3]. However, the sex distribution is equal, except in the Middle Eastern population, where males are more affected. Simultaneously, females are more commonly affected in Southeast Asian countries, such as Japan and Korea [4, 5]. Moreover, there is a strong relationship between the geographic distribution of BD prevalence and the human leukocyte antigen (HLA)-B51, with a positive gene frequency of 50-80% along the Silk Road countries, dropping to 2-8% in the Northern European countries and the United States of America. The prevalence of HLA-B51 positivity is 76.9% in Saudi patients with BD [3]. Currently, several diagnostic criteria for BD are used globally. One of these is the revised diagnostic criteria proposed by the Behçet’s Disease Research Committee of Japan, which is the only criterion that involves gastrointestinal manifestations [6]. Another one is the 1990 International Study Group for Behçet’s disease criteria, which includes major and minor criteria. Lastly, the most recent among the criteria is that of the International Criteria for Behçet’s Disease. These criteria include a scoring system for multiple clinical manifestations, with a score of 4 indicating a BD diagnosis [7]. Nevertheless, the clinical assessment of disease activity in patients with BD is challenging. This can be attributed to several factors, including exacerbation and remission. Targeted remission is insufficient, and relapse should be prevented. Another reason is the multi-organ involvement of BD. Each organ should be assessed and evaluated differently [8]. Saudi Arabia, located in the Silk Road area, is a hotspot for this disease. Limited data about the clinical presentation and patient demographics in this region exist. Hence, this study aimed to fill knowledge gaps and improve understanding of the disease's impacts on patients, its clinical presentations, and typical progression. Specifically, this study aimed to analyze the demographic and clinical characteristics of patients with BD at the King Abdulaziz University Hospital, explore the differences in clinical features according to demographic factors (age, sex, nationality, and body mass index (BMI)), and identify the association between clinical and laboratory findings and disease severity. In addition, this study aimed to help rheumatologists in Saudi Arabia to better understand the disease. The study findings will provide valuable insights regarding patients with BD, particularly in the Saudi Arabian population.

## 2 MATERIALS AND METHODS

### 2.1 Participants

This was a retrospective core chart review. We included adult patients who visited the rheumatology clinic of King Abdulaziz University Hospital in Saudi Arabia between 2005 and 2023. The inclusion criteria were age ≥18 years and a diagnosis of Behçet’s disease (BD) based on either the international classification criteria or the International Study Group classification criteria. Before the study, approval was obtained from the Research Ethics Committee of King Abdulaziz University (Reference Number 161-24). This research was conducted on humans following the Helsinki Declaration of 1975, revised in 2013. This study received no funding.

### 2.2 Study Design

This study included patient demographics, such as sex (Sager guidelines were followed), age, disease duration, associated inflammatory markers, and comorbidities. The investigations included complete blood count, erythrocyte sedimentation rate (ESR), C-reactive protein level, lipid profile, kidney function, coagulation profile, vitamin D level, and liver function tests.

The patients were diagnosed and followed up at King Abdulaziz University Hospital based on the above criteria and were included in the study. Data were extracted from the electronic health systems of the institution.

### 2.3 Statistical Analysis

Following collection, the data were analyzed using IBM SPSS version 27 (IBM Corp. Inc., Armonk, NY). Descriptive statistics, including means and standard deviations, were calculated for continuous variables, while frequencies and percentages were calculated for categorical data. The independent t-test was used to compare two groups, while one-way ANOVA with post hoc Least Significant Difference tests was used for comparisons involving more than two groups. Welch's t-test was used as an alternative when variances were unequal. Owing to the limited sample sizes in some subgroups, non-significant results were interpreted with caution, as they may reflect insufficient statistical power rather than a true lack of association. Statistical significance was set at *p* < 0.05.

## 3 EXPERIMENTAL

This study is a retrospective analysis of Behçet's disease (BD) patient data collected over 18 years (2005-2023) at King Abdulaziz University Hospital. The methodology examined clinical and demographic characteristics, laboratory parameters, and their associations with disease severity. Data were extracted from electronic health records and included variables such as age, sex, nationality, BMI, clinical manifestations, and laboratory test results (*e.g.*, CRP, hemoglobin, lipid profiles).

## 4 RESULTS

### 4.1 Characteristics of the Patients

As shown in Table **1**, 81 patients with BD qualified for this study. Based on their medical records, patients with BD who attended the rheumatology clinic at the King Abdulaziz University Hospital of Saudi Arabia during the study period were almost equally composed of males (51.9%, 42 patients) and females (48.1%, 39 patients). Most patients (75.3%) were Saudi nationals, and approximately a quarter (24.7%) were non-Saudi nationals. The oldest and youngest patients with BD were 78 and 18 years old, respectively, with mean age and standard deviation (SD) of 38.48 years and ± 12.4, respectively. The heights of the tallest and shortest patients were 180 and 91 cm, respectively, with mean and SD of 160 cm and ± 14.1, respectively. Only 33.8% of the patients had a normal body mass index (BMI) (kg/m^2^). The majority belonged to either the obese (32.4%) or overweight (25%) BMI groups, and only a few (8.8%) were underweight. Most of the patients had types O (46.2%) and A (30.8%) blood. Only 10% of the patients had type B, and very few had types A+ (5.1%), AB (2.6%), B- (2.6%) and O- (2.6%) blood.

### 4.2 Clinical Manifestation of Patients

Upon reviewing the records of the patients with BD, 13 clinical manifestations were observed. As shown in Table **2** and Fig. (**1**), the three most common clinical manifestations were oral ulcers (56.8%, 46 patients), genital ulcers (37%, 30 patients), and uveitis (24.7%, 20 patients). Other less common clinical manifestations included deep venous thrombosis (11.1%), skin lesions (13.6%), and arthritis (22.2%). In addition, unpopular clinical manifestations that were minimally observed in patients with BD include a positive pathergy test (3.7%), large vein thrombosis (2.5%), arterial thrombosis (1.2%), neurological manifestations (1.2%), retinal vasculitis (1.2%), erythema nodosum (1.2%), and ocular lesions (1.2%). Superficial phlebitis was not observed in any patient based on the clinical records.

### 4.3 Laboratory Results of Patients

Based on the investigated medical records, 21 laboratory tests were conducted on patients with BD at the rheumatology clinic of the King Abdulaziz University Hospital. The most common laboratory tests performed in >70 patients were C-reactive protein test (CRP) (77), hemoglobin (HB) test (80), platelet count (80), white blood cell count (WBC) (80), neutrophil count (80), aspartate aminotransferase (AST) level (79), alanine aminotransferase (ALT) level (79), gamma-glutamyl transferase (GGT) level (78), bilirubin level (79), albumin level (78), creatinine level (78), and antinuclear antibody (ANA) level (78). Conversely, erythrocyte sedimentation rate (ESR) (64), partial thromboplastin time (62), prothrombin time (63), international normalized ratio (61), Vitamin D3 level (60), triglycerides level (45), and HBA1C level (42) were conducted in 50%<x<80% of the patients. The less commonly performed laboratory tests for patients with BD were high(HDL) (28) and low (LDL) (33) density lipoprotein levels, respectively (Table **3**).

In the 80 patients, the average HB, platelet count, WBC, and neutrophils were 13.21 g/dL, 286.49 k/uL, 7.46 k/uL, and 6.28 k/uL, respectively. Based on inflammatory marker tests, such as ESR and CRP, the average results among the tested patients were 17.73 mm/h and 12.77 mg/L, respectively, indicating moderate inflammation levels. Regarding liver function tests conducted on>95% of the patients, the average AST, ALT, GGT, bilirubin, and albumin levels were 28.76 (U/L), 62.9 (U/L), 42.96 (U/L), 12.36 (U/L), and 41.04 U/L, respectively, indicating normal to elevated levels. The average lipid profile of the patients was 1.97, 1.39, and 3.13 mmol/dl for triglycerides, HDL, and LDL, respectively. The records demonstrated that patients had an average of 6.32% for HBA1C, 35.30 s partial thromboplastin time, 16.55 s prothrombin time, and 1.29 for international normalized ratio. The patients had an average of 76.78 creatinine (µmol/L) levels. Finally, of the 96% of patients tested, 33% were ANA-positive, while 63% tested negative (Table **3**).

### 4.4 Age-based Comparison of Laboratory Results

The degree of variation in laboratory results based on the age of the patients is presented in Table **4**. To determine this, patients’ ages were grouped into ≤30 years, 31-45 years, and >45 years. The ANOVA results demonstrated that among the different variables tested, only HDL levels demonstrated a statistically significant variation (*p* = 0.013) at a confidence level of 0.05. Furthermore, HDL results of patients with BD ≤30 years were significantly different from those patients aged 31-45 years and >45 years. However, the HDL levels in patients aged 31-45 years were not significantly different from those in patients aged >45 years.

### 4.5 Sex-based Comparison of Laboratory Results

As shown in Table **5**, the statistical analysis revealed that, among the different variables tested, only the HB results demonstrated a statistically significant variation (*p* = 0.001) at a confidence level of 0.05. Further analysis showed that HB levels were significantly higher in males than in females. Moreover, results demonstrated that males had higher mean HB levels than females.

### 4.6 BMI-based Comparison of Laboratory Results

As shown in Table **6**, out of the 22 variables tested, only four demonstrated a significant difference in BMI. Based on the ANOVA results, the CRP, AST, ALT, and GGT levels were significantly different at a confidence level of 0.05. Further analysis revealed that patients with underweight BMI exhibited higher levels of CRP (*p* = 0.004), ALT (*p* = 0.023), AST (*p* = 0.003), and GGT (*p* = 0.034) than those with normal, overweight, and obese BMI.

## 5 DISCUSSION

BD is a rare, multi-systemic disease, and its clinical assessment is challenging. Saudi Arabia is part of the Silk Road, where it is prevalent; however, regional data remain limited. This study was conducted to address this gap by providing additional insights into the clinical manifestations of BD among Saudi patients. It is in our best interests to provide additional information to further describe BD manifestations among Saudi patients.

To analyze the demographic and clinical characteristics of Saudi patients, we reviewed the medical records of patients with BD between 2005 and 2023 (18 years of medical records) at the King Abdulaziz University Hospital and identified 81 cases. This number is less than that in the study conducted by Alharty *et al.* in 2023, wherein the authors identified 91 patients with BD in a single tertiary center in Riyadh, Saudi Arabia, over 6 years (January 2015 to December 2021) [9]. However, this number was greater than that reported by Al Zharani *et al.* in 2019, with 47 patients with BD within a tertiary medical center in Southwestern Saudi Arabia, reviewing medical records spanning over approximately an equal number of years (five years) [10]. A disparity in BD prevalence within different regions of Israel was also reported by Klause *et al.* [11]. One potential reason for this is that specialized centers receive more patient referrals than unspecialized ones, such as the King Abdulaziz University Hospital. In addition, >75% of the patients were Saudi nationals; therefore, the study results can represent the Saudi population.

Although almost similar, more male patients (51.9%) than female patients were identified. Al Zharani *et al.* and Alharty *et al.* reported that >50% of patients with BD were male based on the medical records reviewed in Saudi Arabia [9, 10]. Similarly, Kural-Seyahi *et al.*, Davatchi *et al.*, and Gheita *et al.* reported that >50% of BD cases occurred in men in Turkey, Iran, and Egypt, respectively [12-14].

The patients with BD from the King Abdulaziz University Hospital had a BD onset age of 38.48 ± 12.4 years. These findings were higher than those of Al Zharani *et al.* and Alharty *et al.* [9, 10]. In 2023, Alharty *et al.* reported a BD onset age of 29.6 ± 11.4 years for 91 Saudi patients [9]. Conversely, Al Zharani *et al.* reported a BD onset age of 37.11 ± 11.9 years among 47 patients with BD [10]. Davatchi *et al.*, covering 34 countries with BD cases, reported different average ages of onset, ranging from 21.8 to 35.7 years [15]. However, in 2018, Ishido *et al.* reported a mean onset age of 40 years based on a Japanese nationwide registration [16]. This age coincided with those reported globally.

This study identified that most of the patients with BD who attended the King Abdulaziz University Hospital were overweight. This finding was similar to that of Alharty *et al.* in patients with BD at the National Guard Hospital [9]. Chen *et al.* reported an association between BD and metabolic syndrome [17]. In this study, most patients had either blood type A or O. This finding is similar to that reported by Cildag *et al.* in a blood type association study on rheumatic diseases [18].

Medical records revealed that oral ulcers (56.79%), genital ulcers (34.57%), uveitis (24.69%), skin (13.58%), and arthritis (13.58%) are the most common clinical symptoms of BD. These results are not entirely consistent with those reported for Saudi populations from other regions. In 2018, Al Zharani *et al.* reported that oral and genital ulcerations, arthralgia, and weak vision were the most common clinical manifestations of BD in the Southwestern region of Saudi [10]. In contrast, Alharty *et al.* reported that the frequently observed clinical symptoms of BD in Western Saudi Arabia are oral and genital ulcers, arthritis, and pseudofolliculitis [9]. In addition, in 2017, Davatchi *et al.* published a review article on BD presenting a summary of the clinical manifestations of BD and reported that the most commonly observed symptoms are oral aphthosis (>95%), genital aphthosis (60%-90%), skin conditions, including pseudofolliculitis and erythema nodosum, (40%-90%), and eyes defects, including uveitis and retinal vasculitis (45%-90%) [15]. This study’s results confirmed the common clinical manifestations generally observed in patients with BD.

This study also intended to describe the differences in clinical features by demographic factors namely age, nationality, sex, and BMI of patients and laboratory results. Significant variations were found in the clinical features of patients with BD, such as HDL levels differing significantly by age group (*p* = 0.013) and HB levels being higher in males (*p* < 0.001). CRP and albumin levels were significantly higher (*p* = 0.032) and lower (*p* = 0.005), respectively, in non-Saudi Arabians. Regarding these findings, the importance of HDL, HB, CRP, and albumin levels as inflammation markers in BD diagnosis was established long ago. However, variations among patients are less common, particularly in the Saudi population. Understanding these factors will help medical practitioners improve the current management and handling practices in BD treatment. A few critical points of these clinical features associated with BD were reported by Kim *et al.* in the Korean population. The study reported an increased BD risk in participants with highly variable and low mean HDL levels [19]. Furthermore, in 2013, Holzer *et al.* reported that aging alters HDL composition, characterized by defective antioxidant properties, lower paraoxonase 1 activity, and more rapid uptake by macrophages [20]. The hemoglobin value in BD diagnosis was reported by Zhang *et al.* in 2019. In this study, we observed high HB values during BD diagnosis and determined disease activity in patients with BD [21]. However, in 2015, Attar reported a significant association between increased total cholesterol and high CRP levels and a positive correlation between total cholesterol and disease activity among patients with rheumatoid arthritis in Saudi Arabia [22]. In addition, Jiang *et al.* reported that albumin is negatively associated with CRP levels, and CRP and albumin levels vary based on race and smoking habits [23].

Furthermore, this study aimed to identify the association between demographic features, laboratory findings, and disease severity. We observed that patients’ BMI was significantly associated with disease activity. In addition, the analysis revealed that underweight patients exhibited higher CRP (*p* = 0.004), ALT (*p* = 0.023), and AST (*p* = 0.003) levels, indicating a more active disease or complications.

Many variables from the laboratory results demonstrated no significant differences. This lack of significance could be explained by the smaller subgroup sizes, which may not have been sufficiently large to detect statistical significance. For example, the lack of significance in variables like ESR and ALT may be attributed to the relatively small number of patients in each age group. ESR and CRP showed no significant sex differences, which could be related to the small sample size within each sex group, limiting the detection of significant associations. This could also be similar in ESR and WBC, where no significant differences by nationality were observed, possibly because of the limited sample sizes within the non-Saudi group. Lastly, the non-significant results for other variables, such as ESR and bilirubin, might be due to the small sample sizes of the BMI categories, reducing the statistical power to detect differences.

In summary, this study highlighted the multifaceted nature of Behçet’s disease in the Saudi population and offered a detailed exploration of its demographic and clinical variability. These findings underscore the importance of considering patient-specific factors in disease assessment and management, particularly BMI's impact on disease activity. By bridging gaps in local and regional data, this research enhances our understanding of BD and sets the stage for future investigations aimed at refining diagnostic criteria and optimizing therapeutic approaches. The insights gained can improve patient outcomes and help in developing evidence-based guidelines tailored to regional needs.

This study had a few limitations. First, as a retrospective study, it was inherently limited by the availability and accuracy of the data recorded in the medical records. Certain variables, such as detailed treatment regimens, patient adherence, and long-term outcomes, were not consistently documented, restricting our ability to assess the impact of therapeutic interventions on clinical outcomes.

Second, the study was conducted at a single tertiary center, which may limit the generalizability of the findings to Saudi patients. The referral bias of tertiary centers may have resulted in a higher prevalence of severe cases, potentially skewing the clinical and demographic profiles.

Third, the relatively small sample size, particularly within subgroups, reduced the statistical power to detect significant associations of some variables, such as laboratory parameters across BMI categories or nationality. This limitation should be considered when interpreting the study results.

Fourth, this study included no evaluation of imaging findings or detailed assessment of organ-specific involvement, which could have provided a more comprehensive understanding of disease patterns and severity.

Finally, the study’s exclusion of pediatric cases limited its ability to capture the full BD spectrum, as the disease often presents differently in younger patients. Future studies with larger multicenter cohorts and prospective designs should address these limitations to provide a more robust understanding of BD in Saudi Arabia.

Despite these limitations, this study offers valuable insights into the clinicodemographic characteristics of patients with BD in the Saudi population and highlights key areas for future research and clinical practice improvements.

## CONCLUSION

This study provides a comprehensive overview of the clinicopathological characteristics of patients with BD in a tertiary care center in Saudi Arabia. The nearly equal sex distribution, higher onset age, and common clinical manifestations observed in our cohort align with regional and global trends while reflecting the unique aspects of the Saudi population. This study demonstrated that there are significant associations between demographic factors (age, sex, nationality, and BMI) and laboratory parameters (CRP, albumin, and HDL), underscoring the complex interplay between patient characteristics and disease expression.

Our findings contribute to the growing evidence body necessary to understand BD in the Middle Eastern context and offer insights that can inform the development of tailored management strategies. The observed relationship between BMI and disease activity, particularly in underweight patients, warrants further investigation to optimize the care for this patient subset.

Future studies should focus on larger multicenter cohorts and review pediatric cases to improve the generalizability of the study findings.

## AUTHORS’ CONTRIBUTIONS

YB and MM contributed equally to the study design, data collection, analysis, and manuscript preparation. Both authors reviewed and approved the final version of the manuscript.

## Figures and Tables

**Fig. (1):**
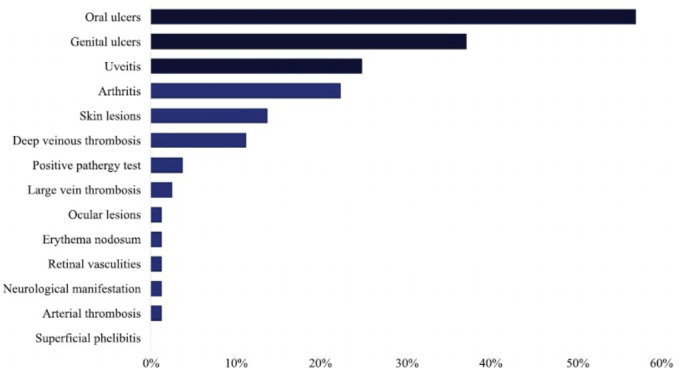
Clinical manifestations among patients with BD (N=81).

**Table 1:** Characteristics of patients with BD.

**Demographics**	**-**	**N**	**Min**	**Max**	**Mean**	**SD**
Age	-	81	18	78	38.48	12.4
Height (cm)	-	68	91.0	180.0	160.07	14.1
Weight (kg)	-	68	10.0	129.0	70.87	20.1
BMI	-	68	12.1	50.4	27.57	7.9
-	-	-	-	**Count**	-	**%**
Total	-	-	-	81	-	100.0
Age	<=30	-	-	19	-	23.5
	31-45	-	-	40	-	49.4
	45>	-	-	22	-	27.2
Sex	Male	-	-	42	-	51.9
	Female	-	-	39	-	48.1
Nationality	Saudi	-	-	61	-	75.3
	Non-Saudi	-	-	20	-	24.7
Diagnosis	Behcet's disease	-	-	81	-	100.0
BMI	Underweight	-	-	6	-	8.8
	Normal	-	-	23	-	33.8
	Overweight	-	-	17	-	25.0
	Obese	-	-	22	-	32.4
Blood type	A	-	-	12	-	30.8
	A+	-	-	2	-	5.1
	AB	-	-	1	-	2.6
	B	-	-	4	-	10.3
	B-	-	-	1	-	2.6
	O	-	-	18	-	46.2
	O-	-	-	1	-	2.6

**Table 2:** Clinical manifestations among patients with BD (N=81).

**Manifestations**	**Count**	**%**
Oral ulcers	46	56.8
Genital ulcers	30	37.0
Uveitis	20	24.7
Arthritis	18	22.2
Skin lesions	11	13.6
DVT	9	11.1
Positive pathergy test	3	3.7
Large vein thrombosis	2	2.5
Ocular lesions	1	1.2
Neurological manifestations	1	1.2
Erythema Nodosum	1	1.2
Arterial thrombosis	1	1.2
Retinal vasculitis	1	1.2
Superficial phlebitis	0	0.0

**Table 3:** Laboratory results of BD patients.

**Test Conducted**	**-**	**N**	**Min**	**Max**	**Mean**	**SD**
ESR	-	64	1.00	102.00	17.73	20.6
CRP	-	77	2.86	184.00	12.77	25.3
HB	-	80	5.32	18.10	13.21	2.2
PLT	-	80	47.00	486.00	286.49	84.4
WBC	-	80	3.22	30.70	7.46	3.6
Neutrophils	-	80	0.71	82.30	6.28	13.7
AST	-	79	7.00	152.00	28.76	25.7
ALT	-	79	7.00	982.00	62.90	113.8
GGT	-	78	4.00	800.00	42.96	96.4
Bilirubin	-	79	2.00	222.00	12.36	24.5
Albumin	-	78	18.00	51.60	41.04	6.0
Triglyceride	-	45	0.29	6.48	1.97	1.4
HDL	-	28	0.57	2.41	1.39	0.6
LDL	-	33	1.40	4.85	3.13	0.9
HBA1C	-	42	4.20	15.00	6.32	2.1
PTT	-	62	11.40	120.00	35.30	15.1
PT	-	63	9.80	120.00	16.55	17.0
INR	-	61	0.85	7.00	1.29	1.1
Creatinine	-	78	27.10	310.00	76.78	39.6
Vitamin D3	-	60	11.71	245.80	65.18	40.4
-	-	-	-	**Count**	**-**	**%**
Total	-	-	-	81	-	100.0
ANA	Positive	-	-	27	-	33.3
	Negative	-	-	51	-	63.0
	N/A	-	-	3	-	3.7

**Abbreviations:** CRP: C- reactive protein, HB: hemoglobin, PLT: platelets, WBCs: white blood cells, AST: aspartate aminotransferase, ALT: alanine aminotransferase, GGT: gamma-glutamyl transferase, HDL: high-density lipoprotein, LDL: low-density lipoprotein, HBA1C: hemoglobin A1C, PTT: partial thromboplastin time, PT: prothrombin time, INR: international normalization rate and VitD3: Vitamin D3

**Table 4:** Statistical analysis on the association between age and laboratory results.

**Age**	**Total**	**<=30**	**31-45**	**45>**	***p*-value**
ESR	64	17.85 ± 19.4	16.43 ± 21.0	20.50 ± 21.7	0.812
CRP	77	9.97 ± 13.1	16.92 ± 33.9	7.65 ± 8.8	0.353
HB	80	13.60 ± 2.4	13.11 ± 2.4	13.05 ± 1.7	0.693
PLT	80	294.94 ± 59.7	279.33 ± 98.5	292.59 ± 75.3	0.752
WBC	80	6.94 ± 2.3	7.80 ± 4.7	7.25 ± 2.2	0.676
Neutrophils	80	6.42 ± 12.9	7.53 ± 17.3	3.88 ± 2.0	0.607
AST	79	20.06 ± 11.7	33.62 ± 31.9	27.27 ± 19.6	0.171
ALT	79	44.06 ± 50.1	81.13 ± 155.7	46.00 ± 31.1	0.376
GGT	78	23.76 ± 15.7	59.97 ± 133.1	27.64 ± 26.7	0.298
Bilirubin	79	10.53 ± 7.1	14.64 ± 34.3	9.82 ± 5.2	0.719
Albumin	78	42.34 ± 4.5	39.46 ± 7.0	42.70 ± 4.7	0.076
Triacyglycride	45	2.61 ± 1.3	1.77 ± 1.3	2.14 ± 1.6	0.452
HDL	28	2.11 ± 0.2^A^	1.28 ± 0.6^B^	1.27 ± 0.5^B^	0.013^a,b^
LDL	33	3.59 ± 0.7	3.19 ± 0.7	2.94 ± 1.1	0.403
HBA1C	42	6.60 ± 1.8	6.21 ± 2.1	6.38 ± 2.4	0.932
PTT	62	40.68 ± 26.0	35.17 ± 12.5	31.98 ± 6.7	0.275
PT	63	16.17 ± 7.6	18.36 ± 23.1	14.08 ± 9.2	0.689
INR	61	1.13 ± 0.3	1.47 ± 1.6	1.15 ± 0.7	0.543
Creatinine	78	72.07 ± 13.8	82.31 ± 50.6	71.10 ± 31.1	0.491
Vitamin D3	60	51.55 ± 19.9	62.64 ± 33.5	77.09 ± 55.2	0.223
ER visits	80	1.68 ± 2.3	4.10 ± 6.1	5.09 ± 6.2	0.131
ANA	-	-	-	-	-
Positive	27	4(14.8%)	16(59.3%)	7(25.9%)	0.378
Negative	51	14(27.5%)	23(45.1%)	14(27.5%)	

**Abbreviations:** CRP: C- reactive protein, HB: hemoglobin, PLT: platelets, WBCs: white blood cells, AST: aspartate aminotransferase, ALT: alanine aminotransferase, GGT: gamma-glutamyl transferase, HDL: high-density lipoprotein, LDL: low-density lipoprotein, HBA1C: hemoglobin A1C, PTT: partial thromboplastin time, PT: prothrombin time, INR: international normalization rate and VitD3: Vitamin D3. a-significant using the one-way ANOVA test at <0.05 level. b-Post-Test = LSD. *Capital letters represent the Post-Hoc multiple pairing summary indicator. When two measures share the same letter, they are statistically similar.

**Table 5:** Statistical analysis on the association between biological sex and laboratory results.

**Biological Sex**	**Total**	**Male**	**Female**	***p*-value**
ESR	64	15.44 ± 21.4	20.33 ± 19.7	0.347
CRP	77	16.16 ± 31.4	9.09 ± 15.8	0.222
HB	80	14.07 ± 2.3	12.29 ± 1.8	<0.001^a^
PLT	80	289.61 ± 79.3	283.21 ± 90.4	0.737
WBC	80	8.01 ± 4.3	6.87 ± 2.7	0.165
Neutrophils	80	6.96 ± 14.8	5.55 ± 12.5	0.647
AST	79	26.30 ± 16.4	31.28 ± 32.6	0.392
ALT	79	53.13 ± 46.7	72.92 ± 155.3	0.443
GGT	78	41.98 ± 53.2	44.00 ± 127.9	0.927
Bilirubin	79	10.19 ± 6.0	14.59 ± 34.4	0.427
Albumin	78	41.15 ± 5.3	40.93 ± 6.8	0.874
Triacyglycride	45	1.91 ± 1.1	2.03 ± 1.7	0.771
HDL	28	1.44 ± 0.6	1.34 ± 0.5	0.641
LDL	33	2.91 ± 0.9	3.31 ± 0.8	0.184
HBA1C	42	6.01 ± 1.2	6.58 ± 2.7	0.391
PTT	62	37.29 ± 18.4	32.87 ± 9.6	0.230
PT	63	16.96 ± 14.3	16.06 ± 20.1	0.836
INR	61	1.35 ± 1.2	1.22 ± 1.1	0.663
Creatinine	78	82.29 ± 29.5	70.68 ± 48.0	0.198
Vitamin D3	60	58.10 ± 41.0	72.27 ± 39.2	0.176
ER visits	80	3.24 ± 5.0	4.38 ± 6.1	0.364
ANA	-	-	-	-
Positive	27	11 (40.7%)	16 (59.3%)	0.128
Negative	51	30 (58.8%)	21 (41.2%)	

**Abbreviations:** CRP: C- reactive protein, HB: hemoglobin, PLT: platelets, WBCs: white blood cells, AST: aspartate aminotransferase, ALT: alanine aminotransferase, GGT: gamma-glutamyl transferase, HDL: high-density lipoprotein, LDL: low-density lipoprotein, HBA1C: hemoglobin A1C, PTT: partial thromboplastin time, PT: prothrombin time, INR: international normalization rate and VitD3: Vitamin D3. a-significant using Independent t-test at <0.05 level.

**Table 6:** Statistical analysis on the association between BMI and laboratory results of patients with BD.

**BMI**	**Total**	**Underweight**	**Normal**	**Overweight**	**Obese**	***p*-value**
ESR	55	8.25 ± 5.2	14.00 ± 17.4	17.08 ± 22.9	17.95 ± 16.7	0.747
CRP	65	49.44 ± 74.6^A^	9.66 ± 15.8^B^	10.18 ± 15.6^B^	7.18 ± 6.3^B^	0.004^a,b^
HB	67	11.62 ± 2.9	13.51 ± 2.0	13.77 ± 1.6	13.16 ± 2.0	0.150
PLT	67	243.67 ± 108.9	281.68 ± 87.7	317.82 ± 76.7	296.64 ± 70.5	0.248
WBC	67	10.09 ± 11.0	6.50 ± 2.4	7.59 ± 2.2	7.83 ± 2.3	0.230
Neutrophils	67	3.13 ± 3.3	3.30 ± 2.0	4.06 ± 2.2	4.16 ± 1.9	0.458
AST	67	52.50 ± 53.0^A^	24.50 ± 12.0^B^	23.76 ± 9.3^B^	22.23 ± 6.9^B^	0.003^a,b^
ALT	67	204.50 ± 383.6^A^	41.50 ± 25.1^B^	46.71 ± 34.2^B^	62.95 ± 55.0^B^	0.023^a,b^
GGT	66	163.17 ± 313.5^A^	36.23 ± 35.7^B^	28.29 ± 29.0^B^	38.00 ± 63.5^B^	0.034^a,b^
Bilirubin	67	11.60 ± 5.1	10.68 ± 6.5	8.91 ± 5.6	8.80 ± 4.0	0.488
Albumin	66	36.92 ± 12.7	41.57 ± 4.6	42.84 ± 4.1	40.76 ± 4.7	0.172
Triglyceride	40	1.22 ± 1.0	1.94 ± 1.7	2.23 ± 1.6	2.07 ± 1.1	0.825
HDL	24	1.57 ± 0.0	1.29 ± 0.5	1.17 ± 0.4	1.37 ± 0.6	0.865
LDL	28	3.14 ± 0.0	2.89 ± 0.6	3.02 ± 1.3	3.06 ± 0.8	0.968
HBA1C	38	5.63 ± 0.3	6.29 ± 2.7	5.87 ± 0.7	7.09 ± 2.7	0.570
PTT	54	42.55 ± 15.0	33.70 ± 7.7	41.49 ± 26.1	28.59 ± 5.4	0.054
PT	54	24.65 ± 28.3	13.32 ± 2.7	15.43 ± 11.8	13.52 ± 6.6	0.182
INR	53	2.04 ± 2.4	1.08 ± 0.2	1.27 ± 0.9	0.99 ± 0.1	0.095
Creatinine	66	89.32 ± 78.4	80.26 ± 30.5	66.06 ± 17.4	81.91 ± 52.8	0.576
Vitamin D3	53	88.50 ± 62.9	77.78 ± 58.5	53.93 ± 22.3	57.05 ± 30.4	0.269
ER visits	67	5.83 ± 4.3	2.65 ± 3.6	3.35 ± 4.8	5.43 ± 6.4	0.221
ANA	-	-	-	-	-	-
Positive	25	2 (8.0%)	8 (32.0%)	5 (20.0%)	10 (40.0%)	0.696
Negative	41	4 (9.8%)	14 (34.1%)	12 (29.3%)	11 (26.8%)	

**Abbreviations:** CRP: C- reactive protein, HB: hemoglobin, PLT: platelets, WBCs: white blood cells, AST: aspartate aminotransferase, ALT: alanine aminotransferase, GGT: gamma-glutamyl transferase, HDL: high-density lipoprotein, LDL: low-density lipoprotein, HBA1C: hemoglobin A1C, PTT: partial thromboplastin time, PT: prothrombin time, INR: international normalization rate and VitD3: Vitamin D3. a-significant using the one-way ANOVA test at <0.05 level. b-Post-Test = LSD. *Capital letters represent the Post-Hoc multiple pairing summary indicator. When two measures share the same letter, they are statistically similar.

## Data Availability

Data used in this study are available upon request from the corresponding author.

## LIST OF ABBREVIATIONS

ALT: Alanine Aminotransferase
AST: Aspartate Aminotransferase
BD: Behçet’s Disease
BMI: Body Mass Index
CRP: C-Reactive Protein
GGT: Gamma-glutamyl Transferase
HB: Hemoglobin
HBA1C: Hemoglobin A1C
HDL: High-density Lipoprotein
INR: International Normalization Rate
LDL: Low-density Lipoprotein
PLT: Platelets
PT: Prothrombin Time
PTT: Partial Thromboplastin Time
SD: Standard Deviation
WBCs: White Blood Cells

## STANDARDS OF REPORTING

SAGER and STROBE guidelines were followed.
